# The Okumus Incision: A Hairline-Preserving Temporozygomatic Approach for Facelift Surgery

**DOI:** 10.1007/s00266-026-06114-5

**Published:** 2026-06-30

**Authors:** Ayhan Okumuş

**Affiliations:** Faculty of Health Science, Mudanya University, Bursa, Turkey

**Keywords:** Face-lift, Modified hairline preserving incision, Okumuş incision, Temporal region, Hairline distortion, Sideburn irregularity

## Abstract

**Background:**

This study aimed to investigate safety and effectiveness of using a modified hairline preserving incision (the Okumus incision) in face-lift surgery, particularly in terms of temporal region aesthetic outcome.

**Methods:**

A total of 400 female patients (mean age: 49 years, range 38 to 72 years) who underwent rhytidectomy using modified hairline preserving incision (the Okumuş incision) through a temporozygomatic approach were included in this study. The Okumus incision was evaluated in terms of objective assessment by the investigator using the Global Aesthetic Improvement Scale (GAIS), patient-reported outcome (appearance after treatment, satisfaction with the treatment), complications and temporal region aesthetic outcome (temporal hairline distortion, anterior temporal scarring, alopecia, or sideburn irregularity).

**Results:**

At postoperative month 12, GAIS scores indicated exceptional improvement in 98% of patients and 98% of patients reported that they were very satisfied with the treatment in terms of temporal region aesthetic outcome. No cases of temporal hairline distortion, sideburn irregularity, or visible anterior temporal scars were observed. All complications including widening of temporal scalp scars (4.2%), delayed wound healing (5.2%) and hypertrophic scarring (2.7%) resolved without long-term functional or aesthetic sequelae.

**Conclusion:**

The Okumus incision offers a hairline-preserving, cosmetically superior alternative for female patients undergoing facelift, without compromising safety or efficacy. Providing a unique balance between the comprehensive exposure of a classical facelift and the aesthetic advantages of short-scar techniques, it enables retention of the full surgical access for cheek and midface rejuvenation while reliably preserving the temporal hairline and avoiding the stigmata of visible temporal scars.

**Level of Evidence IV:**

This journal requires that authors assign a level of evidence to each article. For a full description of these Evidence-Based Medicine ratings, please refer to the Table of Contents or the online Instructions to Authors  www.springer.com/00266.

**Supplementary Information:**

The online version contains supplementary material available at https://doi.org/10.1007/s00266-026-06114-5.

## Introduction

Facelift, or rhytidectomy, is a surgical procedure used to rejuvenate and reshape the face by reversing visible signs of aging through lifting and tightening the skin and deeper facial tissues [1–3].

Owing to rising popularity of rhytidectomy with increasing life expectancy, surgeons’ techniques and approaches have undergone significant advancements over time, as evolved from initial skin-only procedures to superficial musculoaponeurotic system (SMAS) techniques and more complex dual-plane strategies [2–5]. SMAS facelift techniques, as heterogeneous techniques with many variations and modifications, can be divided into three broad categories with comparable aesthetic outcomes including superficial (subcutaneous), SMAS elevation (high SMAS, SMAS flap/extended SMAS, deep plane and composite) and SMAS manipulation (SMASectomy, SMAS plication, and SMAS stacking) procedures [6, 7].

There is no evidence for the superiority of one technique over another, and thus no singular universal approach exists for optimal outcomes [6, 7]. To achieve a natural and long-lasting facelift outcome with high patient satisfaction, advancements in the incision strategy with modifications to avoid visible incision lines are as important as advancements in the surgical technique for improved neckline, jawline and midface [6, 8, 9]. Thus, facial rejuvenation surgery has evolved through numerous modifications of incision design, dissection planes, and fixation techniques, all aiming to optimize procedural safety and aesthetic outcomes while minimizing visible scarring [3, 9].

In 2023, we have reported favorable outcomes of a local anesthesia–based extended superolateral cheek lift with a short preauricular scar, in 200 patients with moderate degrees of facial aging [10]. In that study, we have emphasized the feasibility and safety of the technique, particularly under local anesthesia, and highlighted its applicability for malar, nasolabial, and jawline rejuvenation. Although the incision design was briefly described as a reverse “V” within the sideburn area, the focus of that publication was primarily on the cheek lift technique itself rather than the incision design [10].

Over the past several years, however, we have systematically applied a refined version of this incision not only for isolated cheek lifts, but also for full facelifts in approximately 400 patients This approach, which we now designate as the “Okumuş incision,” represents a hairline-preserving incision modification based on situating the superior limb of the incision entirely within the temporal scalp, addressing the visible and deforming temporal scars as one of the most frequent aesthetic concerns of rhytidectomy specific to management of temporal region [8, 9].

Herein, we present our extensive clinical experience with this hairline-preserving incision modification (the Okumus incision) for facelift surgery, by primarily focusing on the incision itself regarding its ability to address the temporal region problems (sideburn and anterior hairline contour irregularities, visible scars) without compromising the exposure and redraping capacity of a classical facelift.

## Materials and Methods

### Study Population

A total of 400 female patients (mean age: 49 years, range 38 to 72 years) who underwent rhytidectomy (isolated cheek lift: *n *= 250, full facelift: *n* = 150) using modified hairline preserving incision (the Okumuş incision) through a temporozygomatic approach between March 2019 and September 2025 were included in this study. Male patients were excluded because the modification alters the beard line and sideburn, which may compromise natural aesthetics in men.

The study was conducted in accordance with the ethical principles stated in the “Declaration of Helsinki.” Written informed consent was obtained from each subject for the operative procedures and for the use of patient data for publication purposes.

### Assessments

The Okumus incision was evaluated in terms of objective assessment by the investigator using the global aesthetic improvement scale (GAIS), the patient-reported outcome (appearance after treatment, satisfaction with the treatment), the complications and the temporal region aesthetic outcome (temporal hairline distortion, anterior temporal scarring, alopecia, or sideburn irregularity).

Objective assessment was based on the GAIS, which is a 5-point scale (worse, no change, improved, much improved and very much improved) used to rate global aesthetic improvement in appearance compared to pretreatment, as judged by the investigator.

Patient-reported outcome assessment was based on subjective evaluation of appearance after treatment and satisfaction with the treatment using self-reported items prior to the investigator’s evaluation. The appearance after treatment was assessed using a 5-point scale [very much improved (1), much improved (2), slightly improved (3), no change (4), or worsened (5)], while satisfaction with the treatment was assessed particularly in terms of temporal region aesthetic outcome using a three-point scale (very satisfied, satisfied, or not satisfied). Finally, the patients were asked whether they would recommend the treatment to friends and acquaintances.

### Operative Technique

Operations were performed under local anesthesia in 275 patients using lidocaine (Jetokain®, 2 vials for isolated cheek lifts, 4 vials for full facelifts) containing 1/200.000 adrenalin plus marcaine (5 mL) in saline (100 mL for isolated cheek lift operations, 200–300 mL for full facelifts depending on concomitant liposuction), while general anesthesia was used in 125 patients.

**Fig. 1:**
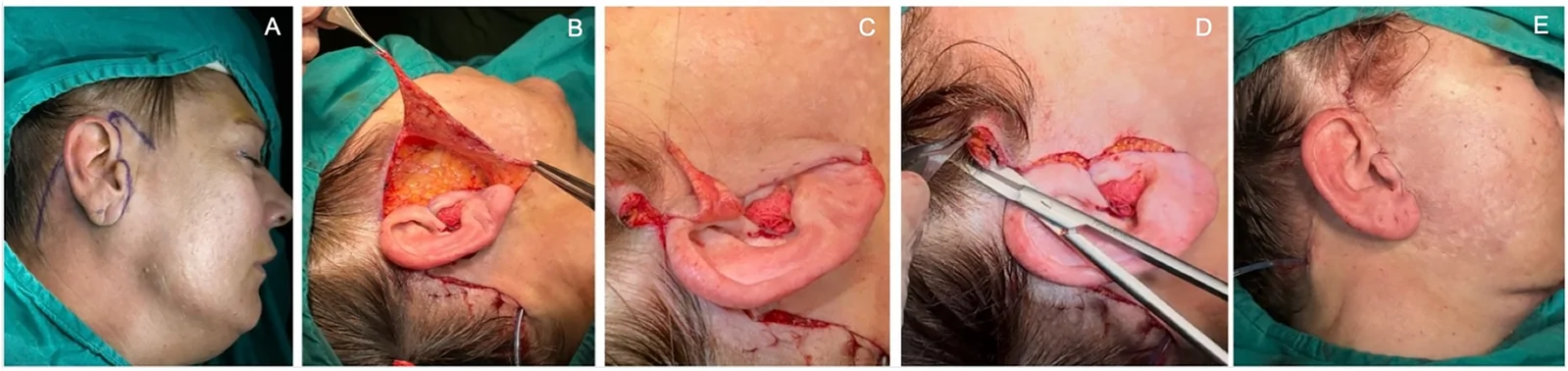
Operative technique for the “Okumuş Incision” based facelift surgery using a hairline-preserving temporozygomatic approach. **A** Skin marking for Okumus incision which starts with the standard preauricular incision line, extending along the natural crease anterior to the tragus and then was directed obliquely (3–4 cm) along the posterior border of the sideburn, at an angle of approximately 45° toward the oral commissure. **B** Skin redrapping after subcutaneous dissection, with upward and oblique traction applied from the distal point of the oblique extension toward the root of the helix and superior hairline. **C** Fixation of flap, as anchored with polydioxanone (PDS) sutures, to deep temporal fascia within the temporal scalp, **D** Management of the dog-ear deformity created superiorly at the fixation point, **E** Final appearance of temporal region with preservation of the natural temporal hairline and sideburn contour

Skin marking began with a standard preauricular incision line, extending along the natural crease anterior to the tragus. Beyond this point, the design of the Okumuş incision diverged from the conventional approach. Instead of curving superiorly toward the anterior temporal hairline, the incision was directed obliquely along the posterior border of the sideburn, at an angle of approximately 45° toward the oral commissure (Fig. 1A). The length of this oblique extension averaged 3 cm but was adjusted intraoperatively depending on the amount of redundant skin to be excised; in cases of greater laxity, the incision could be extended to 4–5 cm. For full facelift procedures, the preauricular and retroauricular portions of the incision proceeded in the classical fashion (Fig. 1A).

After subcutaneous dissection, skin redraping was performed with upward and oblique traction applied from the distal point of the oblique extension toward the root of the helix and superior hairline (Fig. 1B). The flap was anchored with polydioxanone (PDS) sutures to the deep temporal fascia within the temporal scalp (Fig. 1C). This fixation enabled effective vertical–oblique suspension of the malar and cheek skin, corresponding to the length and vector of the oblique incision (Video I, Video II).

A specific feature of this technique is the management of the dog-ear deformity created superiorly at the fixation point. Instead of correcting this redundancy anteriorly—which in conventional techniques results in visible scars or distortion of the temporal hairline—the excess was excised through a secondary incision hidden entirely within the posterior temporal scalp. To facilitate this maneuver, the temporal scalp was widely undermined over the deep temporal fascia, allowing tension-free removal of the dog-ear without hairline irregularities (Fig. 1D, Video III).

The redundant skin inferiorly, in the preauricular region, was excised together with the standard removal of lax cheek and neck skin, as in conventional rhytidectomy (Fig. 1D). In female patients with excessive preauricular hair, the upward redraping allowed selective trimming of undesired hairs that sometimes obscured the auricle, while the remaining hair contributed to concealment of the incision.

In this manner, the Okumuş incision preserves the natural temporal hairline and sideburn contour while providing the exposure and redraping capacity of a classical facelift, achieving effective cheek and midface suspension without visible scarring in the anterior temporal region (Fig. 1E).

## Results

### Clinical Outcomes

**Fig. 2:**
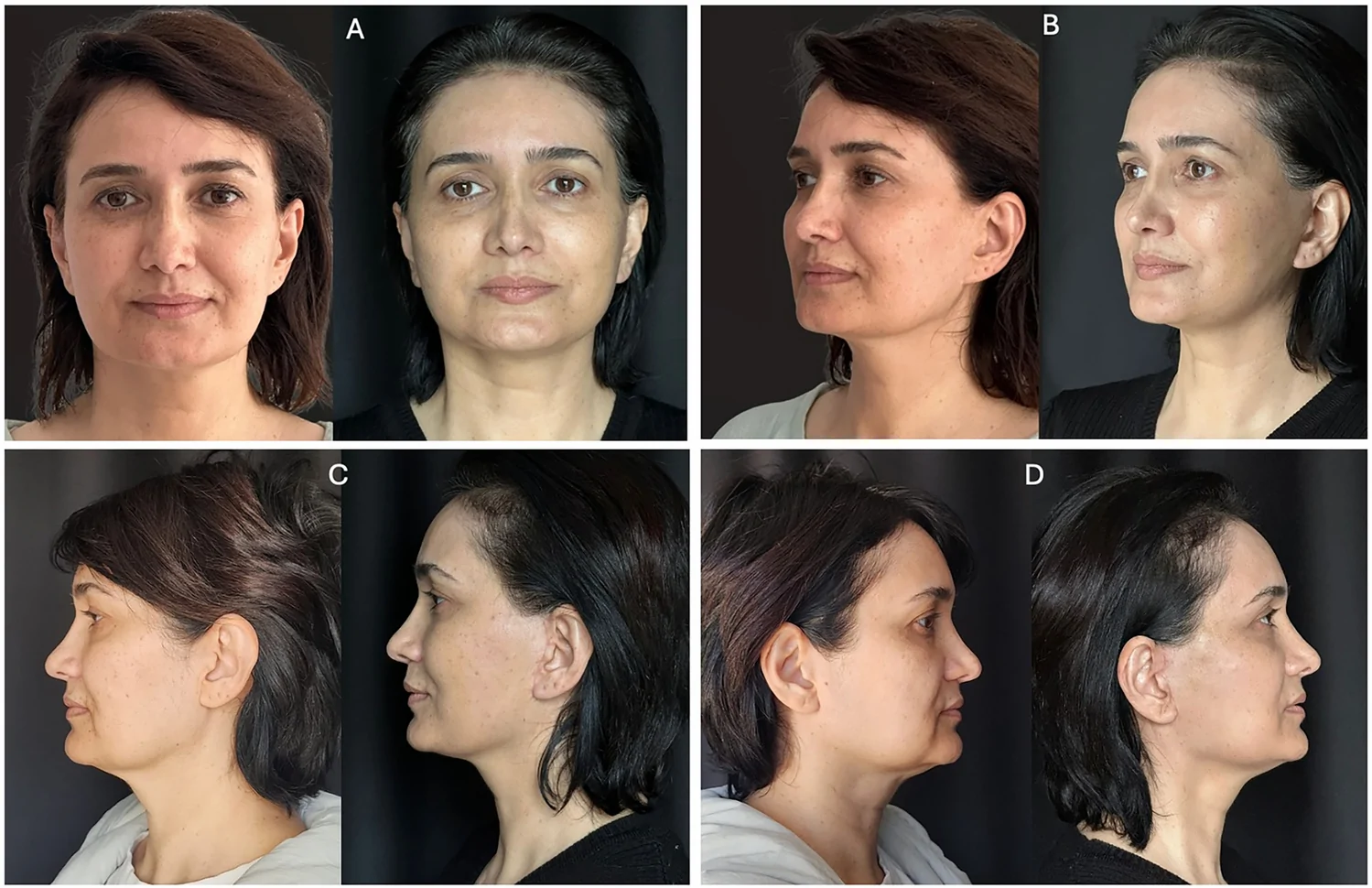
Preoperative (on the left) and postoperative 12th month (on the right) **A** anterior, **B** left oblique, **C** left lateral and **D** right laterale views of a patient

**Fig. 3:**
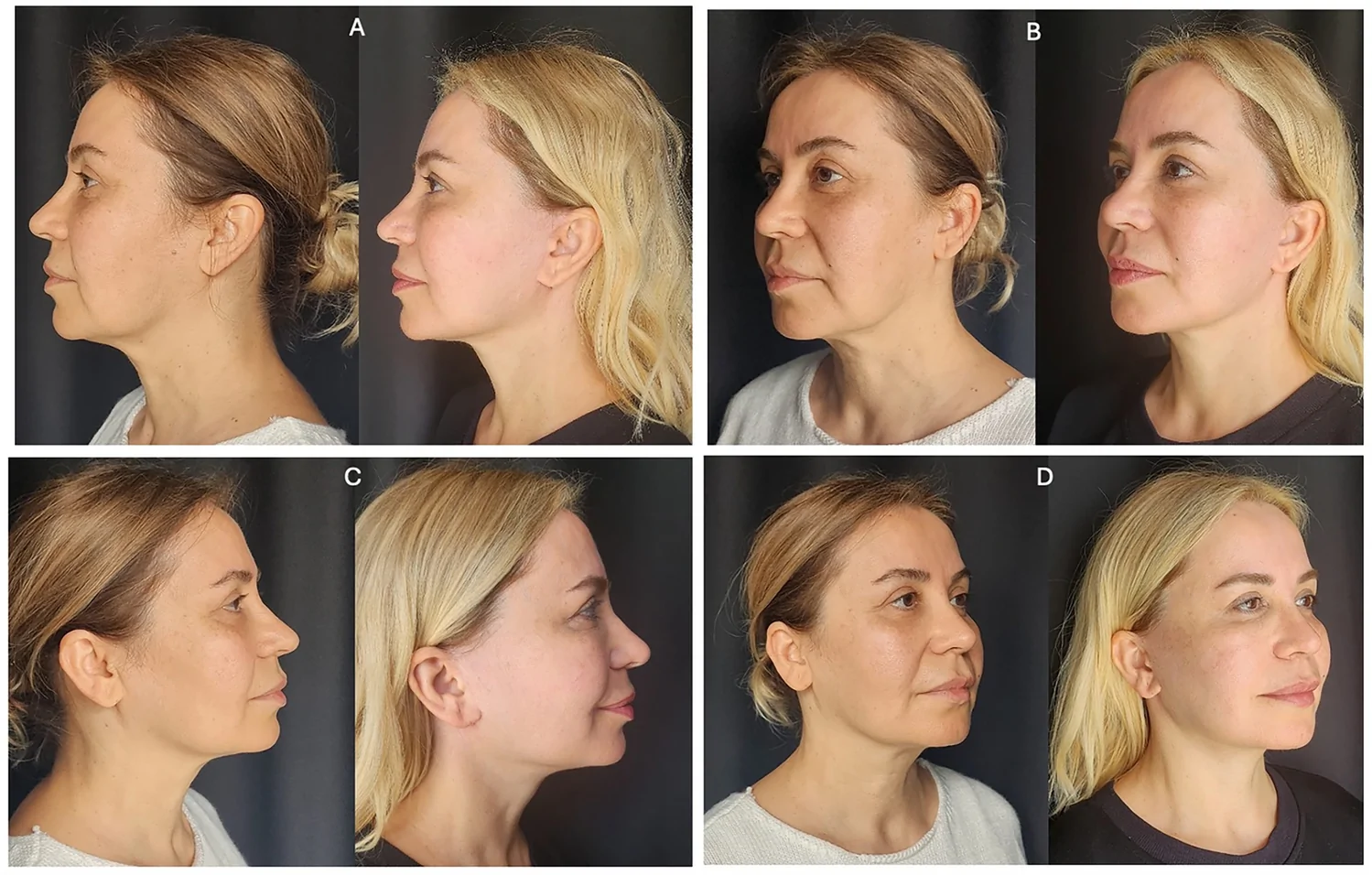
Preoperative (on the left) and postoperative 12th month (on the right) **A** left lateral, **B** left oblique, **C** right lateral and **D** right oblique views of a patient

**Fig. 4:**
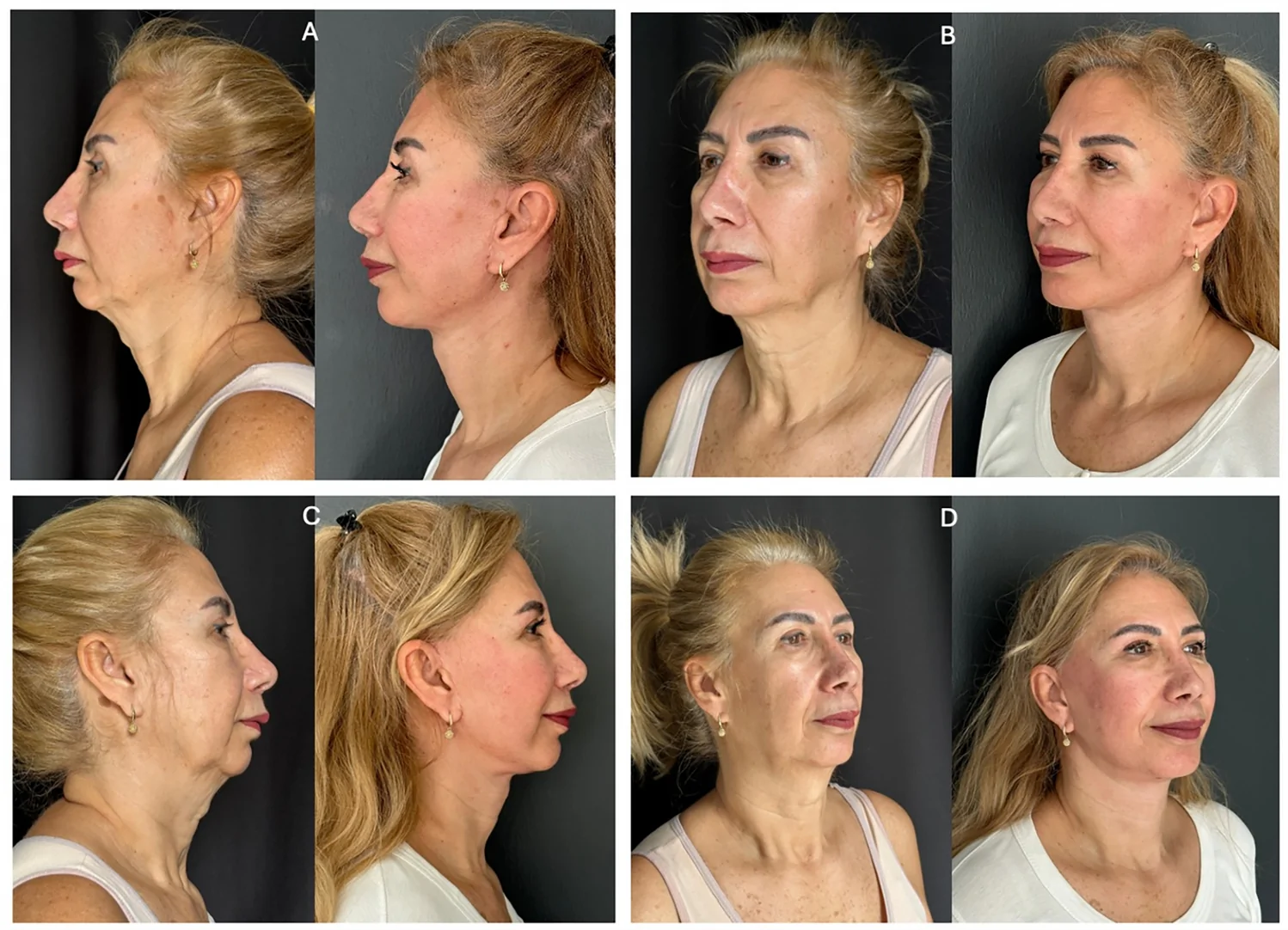
Preoperative (on the left) and postoperative 12th month (on the right) **A** left lateral, **B** left oblique, **C** right lateral and **D** right oblique views of a patient

**Fig. 5:**
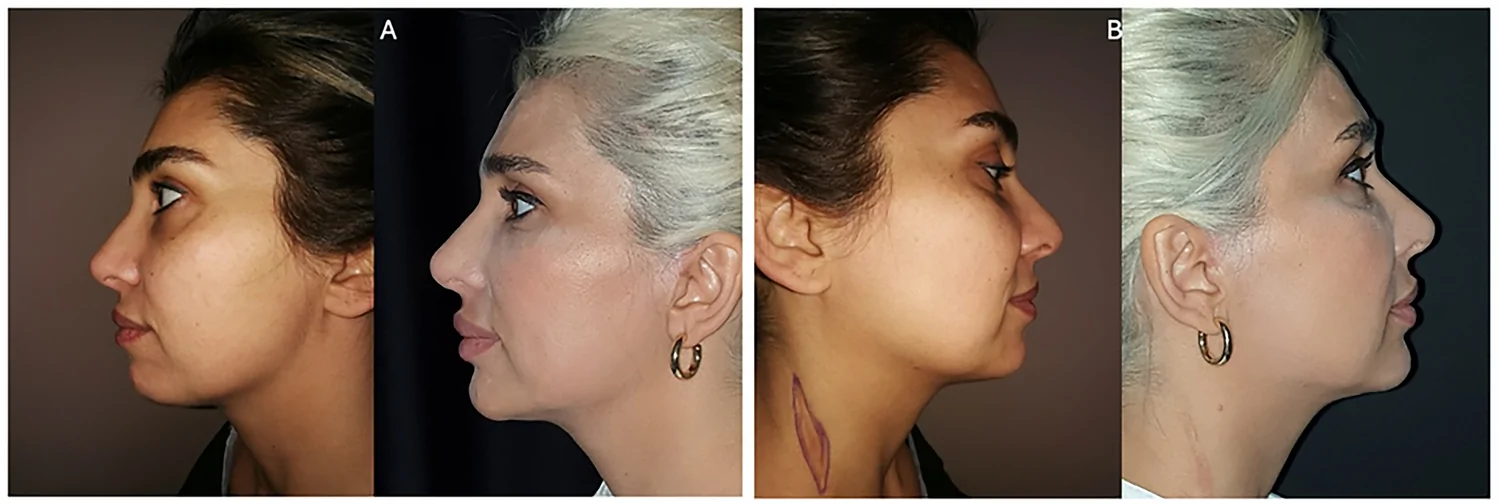
Preoperative (on the left) and early postoperative (on the right) **A** left lateral, **B** right lateral views of a patient

**Fig. 6:**
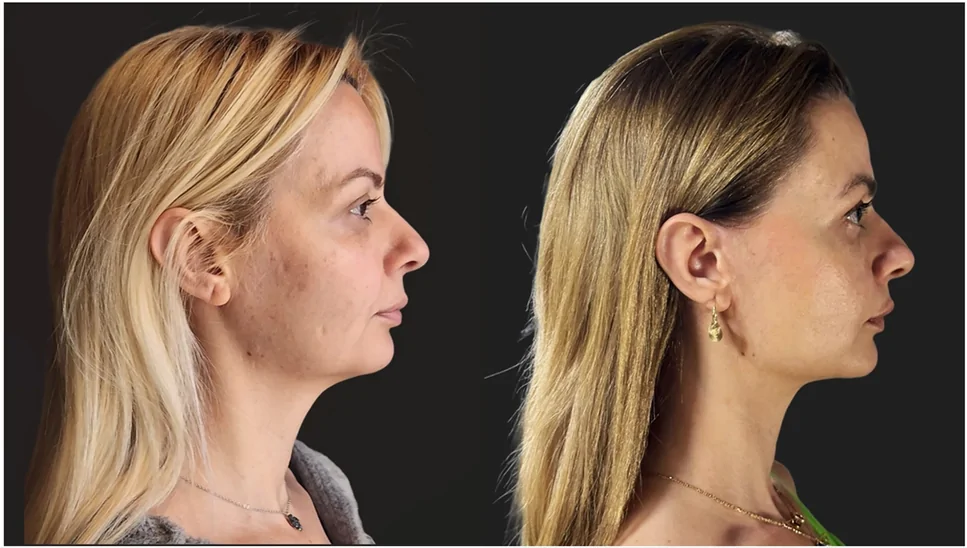
Preoperative (on the left) and early postoperative (on the right) lateral views of a patient

GAIS scores revealed exceptional improvement in 96% of patients at month 1, 97% at month 6 and 98% at months 12. None of the patients was considered to be unaltered or worsened postoperatively (Fig. 2, Fig. 3, Fig. 4, Fig. 5, Fig. 6).

### Temporal Region Aesthetic Outcome

**Fig. 7:**
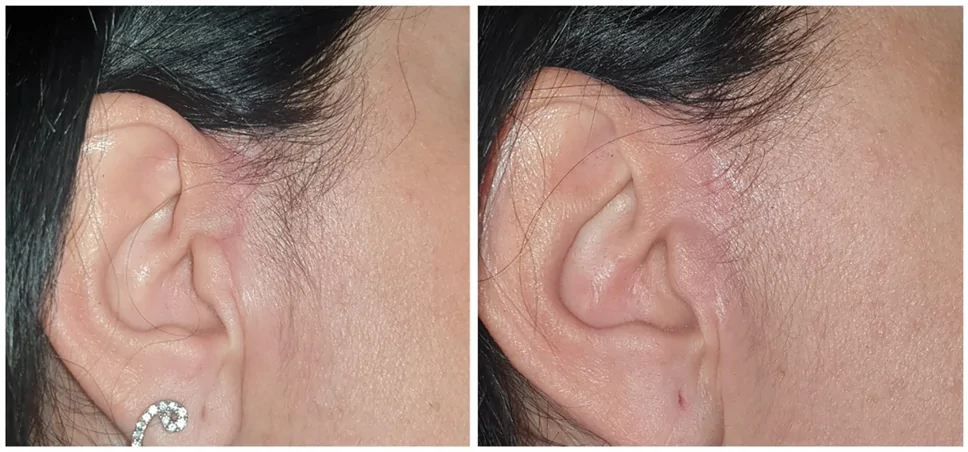
A healed Okumuş facelift incision approximately one month postoperatively. No temporal hairline distortion, sideburn irregularity, or visible anterior temporal scars

No cases of temporal hairline distortion, sideburn irregularity, or visible anterior temporal scars were observed. Because all scars were confined to the preauricular and retroauricular zones, patients achieved a more natural postoperative appearance and faster return to social life (Fig. 7).

Patients experienced less ecchymosis in the temporal region compared with conventional techniques, attributable to the absence of an anterior temporal incision.

### Complications

Complications involved widening of temporal scalp scars in 17 (4.2%) patients, delayed wound healing in 21 (5.2%) patients and hypertrophic scarring (1–3 cm) at upper preauricular or inferior scalp portion of the incision in 11(2.7%) patients. All complications resolved without long-term functional or aesthetic sequelae.

### Patient-Reported Outcome

The appearance after treatment was reported to be very much improved by 95% of patients at month 1, by 98% of patients at month 6 and by 98% of patients at months 12. At postoperative month 12, 98% of patients reported that they were very satisfied with the treatment in terms of temporal region aesthetic outcome and that they would recommend this treatment to friends and acquaintances.

## Discussion

Our findings revealed that the Okumus incision, a modified hairline preserving facelift incision situating the superior limb of the incision entirely within the temporal scalp, represents a successful rhytidectomy technique in terms of maintenance of natural temporal hairline and sideburn contour with elimination of visible scars in the temporal region and the retention of the surgical access necessary for effective rejuvenation. In this regard, Okumus incision offers a hairline-preserving and cosmetically superior alternative for female patients undergoing facelift surgery without compromising safety or efficacy, as associated with favorable postoperative outcomes, low complications rates and a high rate of patient satisfaction.

Although face-lift surgery can be performed with a variety of seemingly different procedures, they all basically involve the steps of incision, undermining, and redraping the skin, and none of the techniques can claim absolute superiority over the other [6, 7, 11, 12]. There is extensive body of literature describing the various rhytidectomy techniques for improving the quality of the face-lift to achieve improved neckline, jawline and midface [8, 9]. However, independent of the technique, improving facial appearance while minimizing the signs of a surgical procedure (i.e., making face-lift incisions as invisible as possible, not altering hairlines to the point of apparent hair loss) is considered a primary goal in face lifting [8, 9, 12]. Hence, facelift surgeons continue to revisit and improve facelift surgery both in terms of incision design and surgical technique to achieve consistently excellent outcomes [8, 9, 12].

The typical facelift incision has 4 components including temporal, preauricular, postauricular, and occipital [11]. One of the most frequent esthetic concerns in facelift surgery is the management of the temporal region, particularly the preservation of the anterior hairline and the natural contour of the sideburn [8, 9]. Other signs of a poor face-lift include hypertrophic scarring, visible preauricular and posterior hairline incision lines, tragal foreshortening, loss of earlobe definition with a pixie-ear configuration, migrating postauricular incisions, postauricular webbing or hypertrophy and a posterior hairline distortion [8, 9, 13].

Although extending vertical incision superiorly into the hairline is advantageous in terms of achieving a greater pull in the temporal area without performing a forehead lift, loss of the temporal hair tuft is a major drawback of this approach [8, 9]. Thus, classical temporal extensions, while providing adequate exposure for midface and cheek lifting, are often associated with undesirable sequelae such as visible scarring, temporal hairline distortion, alopecia along the incision line, and sideburn irregularities [3, 8, 9]. While anterior hairline incisions have been developed as alternative strategy to avoid posterior hairline displacement, they frequently result in conspicuous scars, particularly in patients with thin or short hair [3, 8, 9]. Similarly, mini-/short-scar facelift approaches (i.e., S-lift) may reduce the incision length but often compromise the degree of midface and cheek correction [3, 8, 9]. In contrast, the Okumuş incision was specifically designed to eliminate these drawbacks related to aesthetic outcome in the temporal region without compromising the degree of facial rejuvenation. Basically, by redirecting the superior limb of the incision obliquely along the posterior border of the sideburn and managing superior redundancy through a hidden excision in the posterior temporal scalp, the natural hairline is preserved. In our cohort of 400 female patients, no cases of temporal hairline distortion, anterior temporal scarring, alopecia, or sideburn irregularity were observed. This finding highlights the maintenance of a consistently natural postoperative appearance in the temporal region as the primary advantage of the technique. Although mild superior-posterior displacement of the sideburn may occur in selected patients, it was observed that most patients preferred this trade-off over visible temporal scalp scars or temporal hairline distortion. Hence, the technique may represent a variation of temporal incision concepts with a different balance of advantages and trade-offs, particularly regarding the sideburn position.

Accordingly, the Okumuş incision seems to be a good alternative to classical temporal extensions, in terms of eliminating visible temporal hairline scar and minimizing temporal hairline distortion and also to anterior hairline incisions, in terms of avoiding conspicuous scars at the hairline [8, 9].

Moreover, as compared with mini-/short-scar facelifts, it maintains full access for SMAS and effective vertical–oblique suspension of the skin in the facial rejuvenation area in parallel to the length and vector of the oblique incision, allowing long-lasting correction of midfacial laxity.

Most published studies on complications of facelift surgery have reported hematoma, seroma, necrosis, paresthesia, and poor scarring as the common complications [2, 14–16]. Complications encountered in our series, including hypertrophic scarring (2.7%), delayed wound healing (5.2%), and scar widening (4.2%), were not directly related to the design of the Okumuş incision itself. Also, they were qualitatively different from the complications typically associated with conventional temporal or hairline incisions (hematoma 1 to 9% skin necrosis < 1%, infection 0.18 to 0.89%, facial nerve injury 0.5 to 2.5%, hypertrophic scarring 2–5%) [2, 11, 12, 17–19].

In a systematic review of 27 facelift studies in 6086 patients on the complications and long-term outcomes associated with different SMAS facelift technique, SMAS facelift techniques were demonstrated to achieve satisfactory outcomes with low complication rates, and satisfactory long-terms results without the recurrence of facial redundancy [2]. Overall complication rate of SMAS facelift techniques was relatively low (8.33%), while the temporary facial nerve injury (0.85%), hematoma (1.62%), and skin necrosis (0.41%) were the most commonly reported complications [2]. The SMAS flap technique had the highest overall complication rate (13% total, including hematoma 0.3%, infection 0.08%, wound healing issues 0.2%, seroma 0.04%, alopecia 0.5%, and prolonged dysesthesia 0.04%), followed by SMASectomy (9.44% total, including hematomas 3.3%, scarring 0.8%, temporary neuropraxia 0.2%), high lateral SMAS (12% total, including infection 1.4%, hematoma 1.7%, seroma 0.7%, scarring 0.7%) and SMAS plication (2.12% total, including hematoma 0.6%, delayed wound healing 0.2%, scarring 0.3%, and seroma 0.1%) [2].

In our series, complications were entirely confined to the hidden fixation point above the auricle within the posterior temporal scalp, and all were small and transient events completely resolved without sequelae. Notably, none of the complications affected the hairline or the sideburn and thus the related aesthetic outcome. Also, the dog-ear deformity created superiorly at the fixation point was managed by excision of the excess through a secondary incision hidden entirely within the posterior temporal scalp, which allowed tension-free removal of the dog-ear without hairline irregularities.

Another advantage of Okumus incision seems to be the possibility of extending dissection over the zygomatic area to the close proximity of the nasolabial region, enabling easier approach to mid-face and zygomatic area during skin flap elevation through an improved field of vision and thus more effective oblique traction of the skin in these areas (i.e., up to 4 cm upward traction of zygomatic area).

The Okumus incision was also a favorable technique in terms of recovery and patient satisfaction, enabling patients to achieve a more natural look with reduced temporal bruising and earlier social reintegration, by concealing all scars within natural creases around the ear and scalp. This is particularly important since patients seeking rhytidectomy desire not only an improved neckline, jawline, and midface, but also a natural appearance with inconspicuous incision lines not complicated by conspicuous and disfiguring facelift scars and restoration of reference face with rebuilding a close link between image and personality [5, 8, 9].

## Conclusion

In conclusion, the Okumuş incision offers a hairline-preserving, cosmetically superior alternative for female patients undergoing facelift, without compromising safety or efficacy. Providing a unique balance between the comprehensive exposure of a classical facelift and the aesthetic advantages of short-scar techniques, it enables retention of the full surgical access for cheek and midface rejuvenation while reliably preserving the temporal hairline and avoiding the stigmata of visible temporal scars. The utilization of Okumus incision in facelift surgery should be further investigated via studies with longer follow-up periods to better evaluate the effectiveness of this technique over time.

## Supplementary Information

Below is the link to the electronic supplementary material.

Supplementary file1: Video I: Skin redraping after subcutaneous dissection and the flap anchoring with polydioxanone (PDS) sutures to the deep temporal fascia within the temporal scalp. This fixation enabled effective vertical–oblique suspension of the malar and cheek skin, corresponding to the length and vector of the oblique incision.

Supplementary file2 (MP4 1288 KB) Video II: Skin redraping after subcutaneous dissection and the flap anchoring with polydioxanone (PDS) sutures to the deep temporal fascia within the temporal scalp. This fixation enabled effective vertical–oblique suspension of the malar and cheek skin, corresponding to the length and vector of the oblique incision.

Supplementary file3 (MP4 2553 KB) Video III: Management of the dog-ear deformity by excision of the excess through a secondary incision hidden entirely within the posterior temporal scalp. To facilitate this maneuver, the temporal scalp was widely undermined over the deep temporal fascia, allowing tension-free removal of the dog-ear without hairline irregularities.
